# Availability and Use of HIV Monitoring and Early Infant Diagnosis Technologies in WHO Member States in 2011–2013: Analysis of Annual Surveys at the Facility Level

**DOI:** 10.1371/journal.pmed.1002088

**Published:** 2016-08-23

**Authors:** Vincent Habiyambere, Nathan Ford, Daniel Low-Beer, John Nkengasong, Anita Sands, Mercedes Pérez González, Paula Fernandes, Ekaterina Milgotina

**Affiliations:** 1 Department of HIV/AIDS, World Health Organization, Geneva, Switzerland; 2 Centers for Disease Control and Prevention, Atlanta, Georgia, United States of America; 3 GSSHealth, Baltimore, Maryland, United States of America; Centers for Disease Control and Prevention, UNITED STATES

## Abstract

**Background:**

The Joint United Nations Programme on HIV and AIDS (UNAIDS) 90-90-90 targets have reinforced the importance of functioning laboratory services to ensure prompt diagnosis and to assess treatment efficacy. We surveyed the availability and utilization of technologies for HIV treatment monitoring and early infant diagnosis (EID) in World Health Organization (WHO) Member States.

**Methods and Findings:**

The survey questionnaire included 14 structured questions focusing on HIV testing, cluster of differentiation 4 (CD4) testing, HIV viral load (VL) testing, and EID and was administered annually from 2012 to 2014 through WHO country offices, with each survey covering the previous 12-mo period. Across 127 targeted countries, survey response rates were 60% in 2012, 67% in 2013, and 78% in 2014. There were encouraging trends towards increased procurement of CD4 and VL/EID instruments in reporting countries. Globally, the capacity of available CD4 instruments was sufficient to meet the demand of all people living with HIV/AIDS (PLWHA), irrespective of treatment status (4.62 theoretical tests per PLWHA in 2013 [median 7.33; interquartile range (IQR) 3.44–17.75; median absolute deviation (MAD) 4.35]). The capacity of VL instruments was inadequate to cover all PLWHA in many reporting countries (0.44 tests per PLWHA in 2013 [median 0.90; IQR 0.30–2.40; MAD 0.74]). Of concern, only 13.7% of existing CD4 capacity (median 4.3%; IQR 1.1%–12.1%; MAD 3.8%) and only 36.5% of existing VL capacity (median 9.4%; IQR 2.3%–28.9%; MAD 8.2%) was being utilized across reporting countries in 2013. By the end of 2013, 7.4% of all CD4 instruments (5.8% CD4 conventional instruments and 11.0% of CD4 point of care [POC]) and 10% of VL/EID instruments were reportedly not in use because of lack of reagents, the equipment not being installed or deployed, maintenance, and staff training requirements. Major limitations of this survey included under-reporting and/or incomplete reporting in some national programmes and noncoverage of the private sector.

**Conclusion:**

This is the first attempt to comprehensively gather information on HIV testing technology coverage in WHO Member States. The survey results suggest that major operational changes will need to be implemented, particularly in low- and middle-income countries, if the 90-90-90 targets are to be met.

## Abstract 2

Vincent Habiyambere and colleagues use annual surveys administered through WHO country offices to assess HIV testing coverage.

## Author Summary

**Why Was This Study Done?:**

- Global 90-90-90 targets have reinforced the importance of functioning laboratory services to ensure prompt diagnosis and to assess treatment efficacy.
- World Health Organization (WHO) guidelines for the provision of antiretroviral therapy (ART) for people living with HIV emphasize the need to increase access to HIV testing, treatment, and viral load monitoring.
- In order to understand gaps in laboratory capacity to support further ART scale-up, WHO assesses country capacity to provide HIV diagnosis and treatment monitoring tests, how efficiently HIV diagnosis and treatment monitoring technologies are utilized, and the need for scale-up to meet predicted demand.

**What Did the Researchers Do and Find?:**

- We obtained data on the availability and utilization of technologies for HIV treatment monitoring and early infant diagnosis (EID) across 60% of 127 countries surveyed from 2012–2014.
- Globally, the capacity of available CD4 instruments was found to be sufficient to meet the demand of all people living with HIV/AIDS (PLWHA), irrespective of treatment status; however, capacity to measure viral load was inadequate to cover needs in most reporting countries.
- Of concern, only 13.7% of existing CD4 capacity and only 36.5% of existing viral load (VL) capacity was being utilized across reporting countries in 2013. Lack of reagents, equipment not being installed or deployed, maintenance, and staff training requirements were among the reported reasons for underutilization.

**What Do These Findings Mean?:**

- Overall, among the countries surveyed, increasing numbers of CD4 and VL/EID instruments are being procured.
- However, capacity to measure viral load is insufficient to meet current needs, and underutilization of CD4 and VL technology is widespread.
- Major improvements in laboratory capacity utilization are needed in low- and middle-income countries if the 90-90-90 targets are to be met.

## Introduction

The ambitious Joint United Nations Programme on HIV and AIDS (UNAIDS) 90-90-90 targets require coordinated action to ensure that by 2020, 90% of all people living with HIV know their HIV status, 90% of all people diagnosed with HIV infection receive antiretroviral therapy (ART), and 90% of those receiving ART achieve durable viral suppression [1]. Currently, an estimated 16 million people are receiving ART. Rapid ART scale-up has reinforced the importance of strengthening laboratory services now considered as a critical component of a health system to increase access to ART and to improve the quality of treatment and care for people living with HIV/AIDS (PLWHA) [2–4]. The 2015 World Health Organization (WHO) guidelines [2] recommending that ART is prescribed to all people as soon as possible after a HIV-positive diagnosis regardless of CD4 cell count imply a significant rise in the number of people who need to be started and maintained on treatment. Progress towards the 90-90-90 targets will require a significant expansion of HIV testing to diagnose HIV infection and to monitor treatment efficacy in a robust tiered laboratory network [5,6].

Expanding access to treatment requires high-quality HIV testing technologies, including CD4 testing to assess risk of disease progression, viral load (VL) testing to monitor treatment efficacy, early infant diagnosis (EID) to determine HIV-infection status in HIV-exposed children, and other monitoring capabilities within a tiered laboratory network. Technologies that can be used at point of care (POC) provide an important opportunity to expand access to HIV-related testing [7,8]. The availability and utilization of HIV EID and treatment monitoring technologies in many HIV/AIDS endemic countries have not been formally assessed. A detailed analysis is needed if we are to effectively tackle future challenges to ART scale-up. To this end, in 2012, WHO started to conduct annual surveys to assess the availability and the utilization of CD4, VL, and EID testing technologies in WHO Member States. Full and detailed datasets supporting our findings have been provided [9]. We present 3-y survey data and an assessment of trends of instruments available and tests performed, as well as an analysis of potential theoretical capacity versus the demand.

## Methods

### The Survey Tool

An English-language electronic questionnaire survey tool used annually by WHO to assess ART use in WHO Member States was revised to include 14 structured questions on the availability, functionality, and utilization of CD4, VL, and EID laboratory technologies and the market share for different branded technologies. The questionnaire survey is included as S1 Survey Questionnaire.

The 2012 survey covers 1 January 2011 to 31 December 2011, the 2013 survey covers 1 January 2012 to 31 December 2012, and the 2014 survey covers 1 January 2013 to 31 December 2013. Throughout this article, data from the 2012 survey will be referred to as 2011 data, data from the 2013 survey as 2012 data, and data from the 2014 survey as 2013 data.

### Data Collection

Questionnaires were distributed via WHO regional offices to WHO country HIV officers supporting Ministry of Health HIV programme managers, who used data from their national annual reports to complete the survey within 4 mo (April–July of each survey year). Data collection was focused on the public health sector, which includes ministry of health and various nongovernmental organization (NGO) HIV programmes. The study did not cover the private sector. Unlike NGOs, the private sector does not report data to the national HIV programme. Questionnaires were returned by email to WHO’s Department of HIV/AIDS for data cleaning, verification, and analysis.

Surveys were sent to 127 countries, distributed as follows by WHO region:

- all 47 countries in the WHO African Region (AFRO);
- 33 countries in the WHO Region of the Americas (AMRO; Latin America and Caribbean countries: all countries from the Region of the Americas except the United States of America and Canada);
- all 21 countries in the WHO Eastern Mediterranean Region (EMRO);
- 8 high-burden HIV countries in the WHO European Region (EURO);
- all 11 countries in the WHO South-East Asia Region (SEARO);
- 7 high-burden HIV countries in the WHO Western Pacific Region (WPRO).

The completed questionnaires were jointly collected by WHO country offices, the six WHO regional offices (WHO Regional Office for Africa, Brazzaville; WHO Regional Office for the Americas, Washington; WHO Regional Office for the Eastern Mediterranean, Cairo; WHO Regional Office for Europe, Copenhagen; WHO Regional Office for South-East Asia, New Delhi; and WHO Regional Office for the Western Pacific, Manila) and WHO headquarters, Geneva.

### Data Analysis

For the analysis of deployment of existing HIV EID and monitoring technologies, additional data were used. Specifically, numbers of PLWHA were retrieved from the UNAIDS AIDSinfo Online Database [10]. The midestimate of the number of PLWHA was used for the analysis. When data on PLWHA were unavailable from the above source, information was extracted from the latest country report available on the UNAIDS website [11]. If the country report provided the number of PLWHA only for 1 y of interest, that number was used for all 3 reporting years. If the country report provided only the number of diagnosed HIV patients, that number was rounded up to the next 100. Data on the number of PLWHA on ART were provided by responding countries. Testing technologies were categorized as conventional or POC according to definitions applied by the manufacturer.

The lowest published theoretical number of tests that an instrument can perform per technician per day was used to calculate theoretical capacity of CD4 and VL instruments in all countries responding to at least one survey [12–15]. Capacity analyses were based on the assumption that personnel work an average of 8 h per d, 250 d per y. The internationally accepted number of work days (260) was reduced in consideration of national holidays. The average number of national holidays was calculated for a subset of 18 countries and applied to all countries participating in these surveys [16].

Statistical analysis was performed in XLSTAT statistical application for Microsoft Excel (Addinsoft). Data visualization was done using Tableau software (Tableau Software). The *p*-values for the analysis of trends in instrument market share, nonutilization, and maintenance contracting and servicing were calculated using a chi-square test. In the case of rejection of the null hypothesis of multiple proportions equality, the chi-square test was followed by the Marascuilo procedure employed to simultaneously test all possible pairs of proportions and to identify the proportion(s) responsible for the rejection of the null hypothesis. A *p*-value of <0.05 was considered significant.

## Results

### Survey Response Rate

Survey response rates, based on 127 targeted countries, were 60% (76 countries) in 2012, 67% (85 countries) in 2013, and 78% (99 countries) in 2014 (countries that responded to at least one diagnostic survey question). Over the 3 survey years, 55 (43%) countries responded to all three surveys, 35 (28%) countries to two surveys, 25 (20%) countries to one survey, and 9 (7%) responded to none of the three surveys. As each survey covers the previous 12-mo period, the results refer successively to year 2011, 2012, and 2013.

### Availability and Use of CD4 Instruments

Reporting countries have accumulated CD4-testing capacity sufficient to meet WHO recommendation to perform CD4 tests upon HIV diagnosis and every 6 to 12 mo thereafter. CD4 testing is not necessary for a stabilized patient on ART with suppressed VL, if VL testing is available (Table 1) [5,6].

**Table 1:** Number of CD4 instruments and available and utilized CD4 capacity across all reporting countries, disaggregated by WHO region.

	Number of CD4 Conventional Instruments*	Number of CD4 POC Instruments*	Available CD4 Capacity per PLWHA^#^	Available CD4 Capacity per PLWHA on ART^&^	Number of CD4 Tests Performed per PLWHA on ART^§^
**AFRO**					
2011	2,184	606	3.6	14.1	1.3
2012	2,240	875	3.8	12.7	1.1
2013	2,816	1,808	3.7	10.3	1.3
**AMRO**					
2011	63	27	11.1	N/A	N/A
2012	218	10	5.8	14.4	1.4
2013	165	14	5.4	N/A	N/A
**EMRO**					
2011	75	4	8.0	162.0	1.4
2012	113	5	12.7	211.6	2.6
2013	63	0	16.7	169.7	1.8
**EURO**					
2011	27	0	3.6	25.9	2.0
2012	45	0	6.3	30.8	3.1
2013	52	0	9.8	41.1	3.3
**SEARO**					
2011	214	73	5.6	38.3	1.2
2012	284	124	7.9	39.5	1.5
2013	438	53	4.5	11.9	1.8
**WPRO**					
2011	512	0	18.9	57.2	2.6
2012	509	5	18.1	48.6	2.3
2013	654	60	23.0	53.6	2.0
**Total**					
2011	3,075	710	4.6	18.7	1.4
2012	3,409	1,019	5.2	17.6	1.3
2013	4,188	1,935	4.6	12.8	1.4

AFRO, WHO African region; AMRO, WHO Region of the Americas; ART, antiretroviral therapy; EMRO, WHO Eastern-Mediterranean region; EURO, WHO European region; N/A, data not available; PLWHA, people living with HIV/AIDS; POC, point of care; SEARO, WHO South-East Asian region; WPRO, WHO Western Pacific region.

* Based on a subset of 69, 74, and 71 countries reporting the number of instruments in 2011, 2012, and 2013, respectively.

^**#**^ Based on a subset of 66, 71, and 68 countries with data available for number of instruments and number of PLWHA in 2011, 2012, and 2013, respectively.

^**&**^ Based on a subset of 54, 72, and 57 countries with data available for number of instruments and number of PLWHA on ART in 2011, 2012, and 2013, respectively.

^**§**^ Based on subset of 44, 50, 46 countries with data available for number of CD4 tests and number of PLWHA on ART in 2011, 2012, and 2013, respectively.

**Fig 1:**
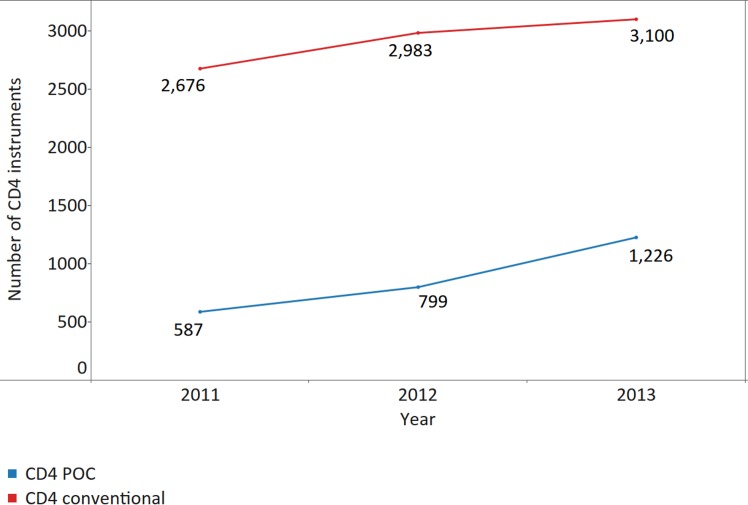
Increase in the number of CD4 instruments reported by 40 countries providing data for 3 consecutive y. Data labels indicate the number of reported instruments. POC, point of care.

**Fig 2:**
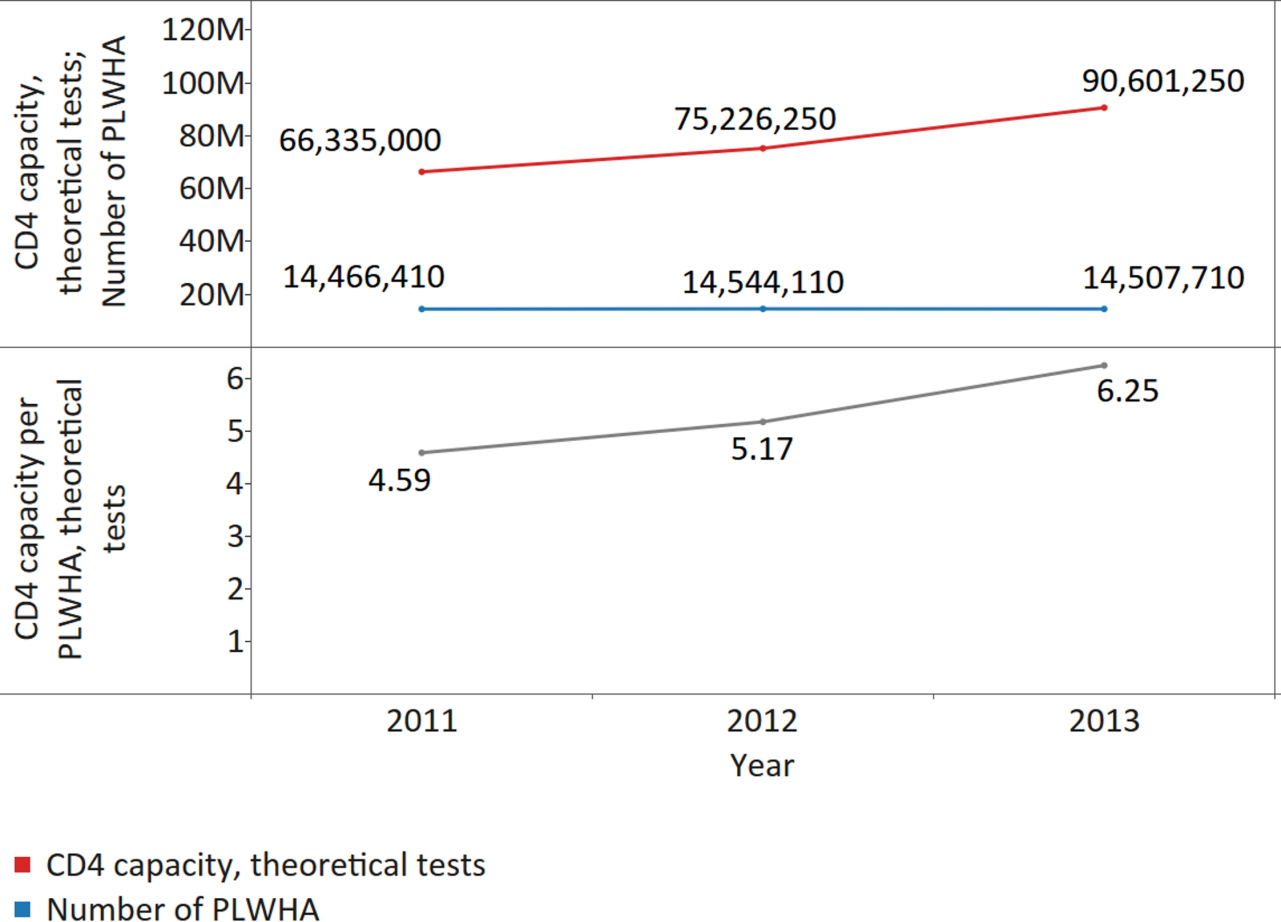
Increase in the theoretical CD4 capacity per people living with HIV/AIDS (PLWHA) across 39 countries providing data for 3 consecutive y. The upper panel shows the number of PLWHA and the theoretical CD4 capacity. The lower panel shows the CD4 capacity per PLWHA. The number of theoretical CD4 tests per PLWHA was calculated for 39 countries, because the number of PLWHA was not available for 1 country.

Across 40 countries reporting CD4 instrument data for 3 consecutive y, the number of CD4 instruments has risen every year between 2011 and 2013, mostly due to the procurement of CD4 instruments for use at POC, which rose from the previous year by 36% in 2012 and by 53% in 2013. Growth for conventional CD4 instruments, however, was only 11% in 2012 and 4% in 2013 in countries responding to all three surveys (Fig 1). As a result, the theoretical capacity of CD4 conventional and POC instruments increased from 66.3 million tests in 2011 to 90.6 million tests in 2013, which equates to a theoretical CD4 testing capacity of 4.59 (median 5.21; IQR 3.07–9.57; median absolute deviation [MAD] 2.56) tests per PLWHA in 2011, 5.17 (median 6.71; IQR 4.10–11.64; MAD 3.31) tests in 2012, and 6.25 (median 7.27; IQR 3.51–11.32; MAD 3.83) tests in 2013 (Fig 2).

Three conventional CD4 instruments, BD FACSCount, CyFlow Counter, and BD FACSCalibur, and only one POC instrument, Alere Pima Analyser, have dominated the CD4 technology market for 3 consecutive y (Table 2). In 2011, 2012, and 2013, 74%, 77%, and 72% of reporting countries utilized WHO prequalified (WHO PQ) CD4 technologies (*X* ^*2*^ [2; 214] = 0.5, *p* = 0.771, effect size [ES] Cramer’s *V* = 0.03).

**Table 2:** The market share by number of branded CD4 instruments in 2011, 2012, and 2013 for all responding countries.

CD4 Platform	CD4 Instrument Brand	WHO PQ Status	2011			2012			2013		
			Number of Countries Reporting	Number of Reported Instruments	Percentage from Total Number of Reported Instruments	Number of Countries Reporting	Number of Reported Instruments	Percentage from Total Number of Reported Instruments	Number of Countries Reporting	Number of Reported Instruments	Percentage from Total Number of Reported Instruments
**Conventional CD4**											
	BD FACSCount	Yes	47	1,676	44.3%	54	1,732	39.1%	48	1,937	31.6%
	CyFlow Counter	No	32	772	20.4%	31	782	17.7%	32	1,018	16.6%
	BD FACSCalibur	No	31	416	11.0%	37	620	14.0%	33	764	12.5%
	EPICS XL	No	10	122	3.2%	12	104	2.3%	16	209	3.4%
	Cytomics FC 500	No	0	0	0.0%	1	1	0.02%	6	84	1.4%
	Millipore Guava	No	11	85	2.2%	14	134	3.0%	12	125	2.0%
	DYNAL MB	No	0	0	0.0%	1	29	0.7%	1	28	0.5%
	Apogee Auto40	No	3	4	0.1%	3	7	0.2%	9	23	0.4%
**POC CD4**											
	Alere Pima	Yes	13	597	15.8%	23	972	22.0%	31	1,874	30.6%
	PointCare NOW	No	4	82	2.2%	6	42	0.9%	5	39	0.6%
	CyFlow miniPOC	No	5	31	0.8%	2	5	0.1%	4	22	0.4%

PQ, prequalification.

**Fig 3:**
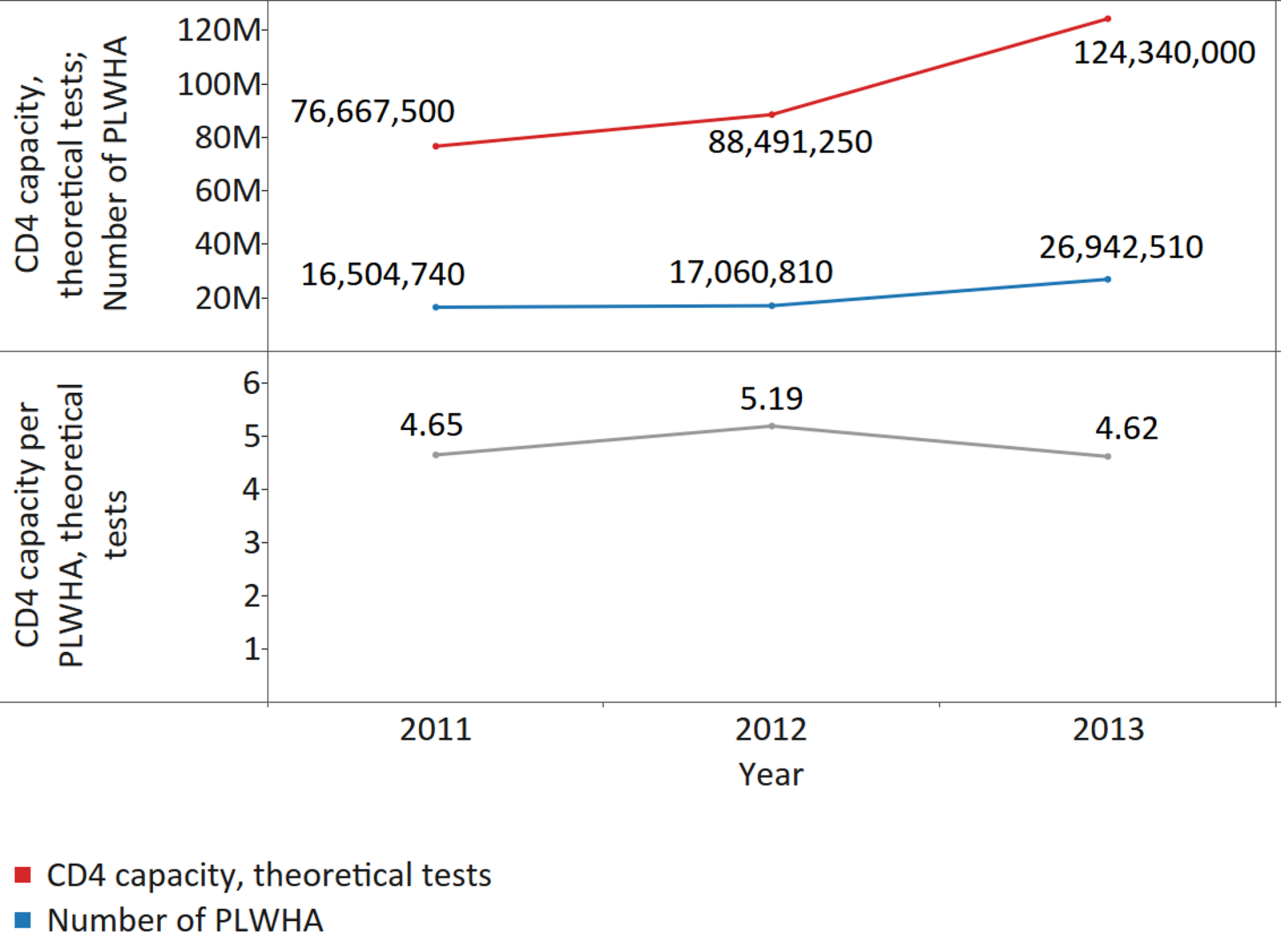
CD4 capacity per people living with HIV/AIDS (PLWHA), for all reporting countries. The upper panel shows the number of PLWHA and the theoretical CD4 capacity for the 66, 71, and 68 countries with data available for number of instruments and number of PLWHA in 2011, 2012, and 2013, respectively. The lower panel shows the CD4 capacity per PLWHA.

All countries with CD4 instruments and with information on number of PLWHA were analysed and the theoretical capacity per PLWHA was measured: there were 66, 71, and 68 countries with data available for number of CD4 instruments and number of PLWHA, respectively in 2011, 2012, and 2013. CD4 capacity was sufficient to cover CD4 testing demand in responding countries, with theoretical CD4 capacity across 68 reporting countries in 2013 of 4.62 tests per PLWHA (median 7.33; IQR 3.44–17.75; MAD 4.35) (Fig 3).

**Fig 4:**
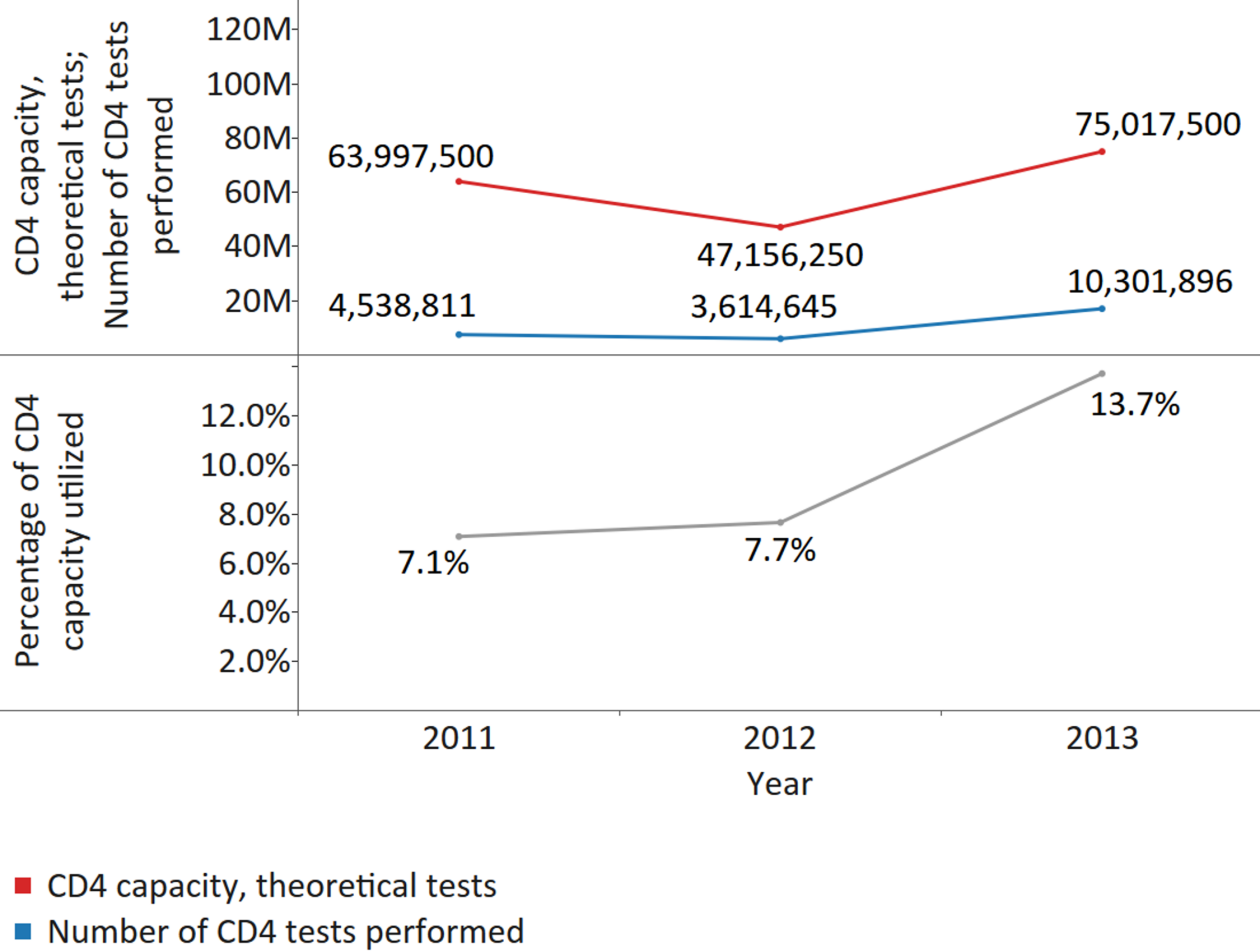
Utilization of CD4 capacity, for all reporting countries. The upper panel displays the number of CD4 tests performed and the theoretical CD4 capacity for countries reporting data on the number of tests performed and the number of CD4 instruments (51, 45, and 50 countries reporting for 2011, 2012, and 2013). The lower panel displays the percentage of CD4 capacity utilized during the reporting period.

All countries that reported the number of CD4 tests performed and the number of CD4 instruments were analysed to calculate the utilization rate of the available theoretical CD4 capacity. CD4 instruments were considerably underutilized, with only 7.1%, 7.7%, and 13.7% of existing CD4 capacity utilized across 51, 45, and 50 countries reporting data on the number of tests done and the number of CD4 instruments in 2011, 2012, and 2013, respectively (*X* ^*2*^ [2; 186,171,250] = 2,062,586, *p* < 0.001, ES Cramer’s *V* = 0.07) (median utilization 3.6% [IQR 0.8%–8.0%; MAD 3.1%] in 2011; 5.9% (IQR 2.6%–12.9%; MAD 4.6%) in 2012; and 4.3% [IQR 1.1%–12.1%; MAD 3.8%] in 2013) (Fig 4). The instrument utilization rate was calculated by dividing the total number of CD4 tests performed by the theoretical CD4 capacity (theoretical number of CD4 tests) for all reporting countries. Suboptimal numbers of CD4 tests were performed per patient on ART per year: on average, 1.44 across 44 countries in 2011 (median 1.25; IQR 0.65–2.07; MAD 0.62), 1.32 across 50 countries in 2012 (median 1.70; IQR 1.13–2.47; MAD 0.72); and 1.41 across 46 countries in 2013 (median 1.54; IQR 0.89–2.26; MAD 0.71). In 2013, 14 (30.4%) countries performed <1 CD4 test per PLWHA on ART, which falls short of current recommendations.

**Fig 5:**
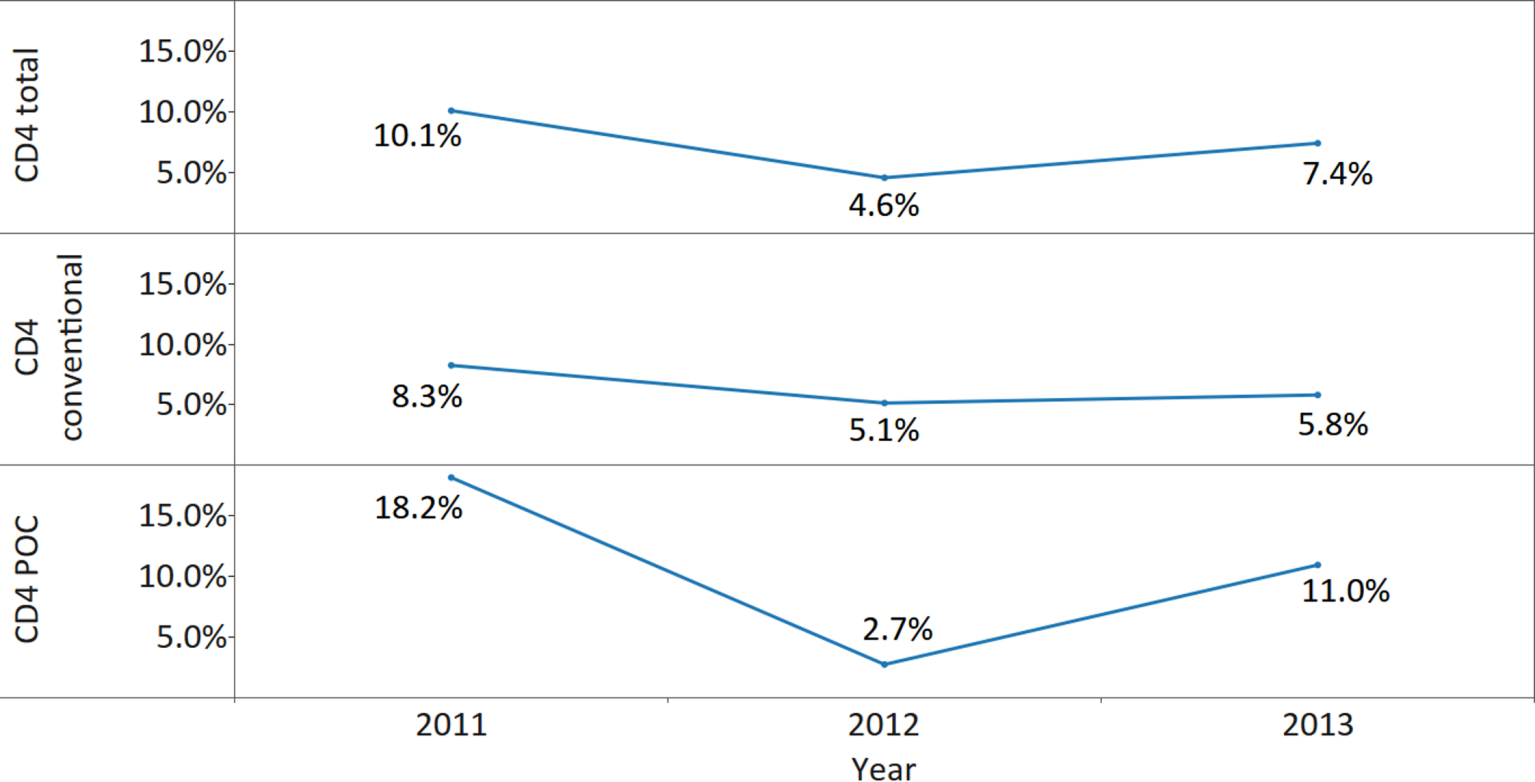
Nonutilization of CD4 instrumentation in reporting countries by survey year and CD4 platform (conventional CD4 versus point of care (POC) CD4). Data labels indicate percentage from the number of all reported CD4 instruments, from CD4 POC, or from CD4 conventional instruments in a particular year.

**Fig 6:**
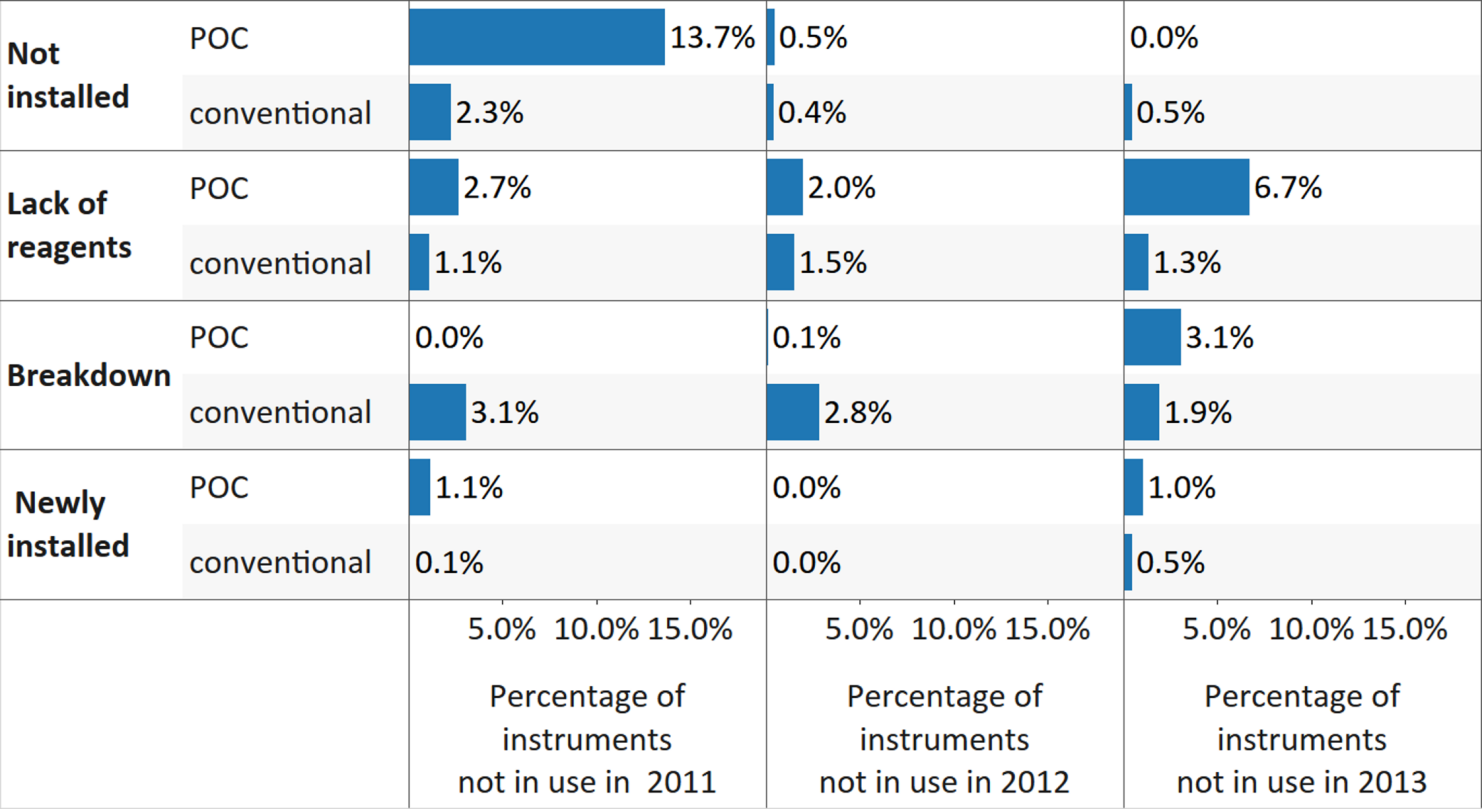
Reasons provided for nonutilization of CD4 instrumentation, for all reporting countries, disaggregated by platform. Data labels indicate the percentage from total number of reported CD4 point of care (POC) or conventional instruments in a particular year.

The surveys highlighted major issues concerning the functionality and use of CD4 instruments. Thirty-seven (53.6%), 33 (44.6%), and 35 (49.3%) countries reported CD4 conventional and/or POC instruments not in use in 2011, 2012, 2013, respectively (*X* ^*2*^ [2; 214] = 1.2, *p* = 0.558, ES Cramer’s *V* = 0.05). In 2013, 455 (7.4%) of all CD4 instruments reported by responding countries were not utilized. The percentage of reported instruments that were currently not being used was significantly higher for CD4 POC than for conventional systems in 2011 (18.2% versus 8.3%; *X* ^*2*^ [1; 3,785] = 62.3, *p* < 0.001, ES *phi* = 0.13) and 2013 (11.0% versus 5.8%; *X* ^*2*^ [1; 6,123] = 51.1, *p* < 0.001, ES *phi* = 0.09) but lower in 2012 (2.7% versus 5.1%; *X* ^*2*^ [1; 4,428] = 10.2, *p* = 0.001, ES *phi* = 0.05), although the ES was negligible in 2012 and 2013 (Fig 5). Key reasons for nonutilization included breakdown for CD4 conventional instruments and lack of reagents for POC CD4 instruments (Fig 6). A number of instruments were reported to be in country but not yet installed, possibly because of lack of technical support, training, and/or reagents, as well as shortages of personnel and lack of deployment planning for newly procured instruments.

While nonutilization of CD4 conventional instruments due to breakdown slightly declined from 2011 to 2013 (*X* ^*2*^ [2; 10,672] = 11.1, *p* = 0.004, ES Cramer’s *V* = 0.02), and nonutilization due to lack of reagents remained mostly unchanged during the same period (*X* ^*2*^ [2; 10,672] = 1.9, *p* = 0.386, ES Cramer’s *V* = 0.01), the situation for POC CD4 instruments worsened from 2011, with breakdown and lack of reagents being the key reasons reported for nonutilization in 2013 (breakdown, *X* ^*2*^ [2; 3,664] = 50.7, *p* < 0.001, ES Cramer’s *V* = 0.08; lack of reagents, *X* ^*2*^ [2; 3,664] = 41.8, *p* < 0.001, ES Cramer’s *V* = 0.07).

**Fig 7:**
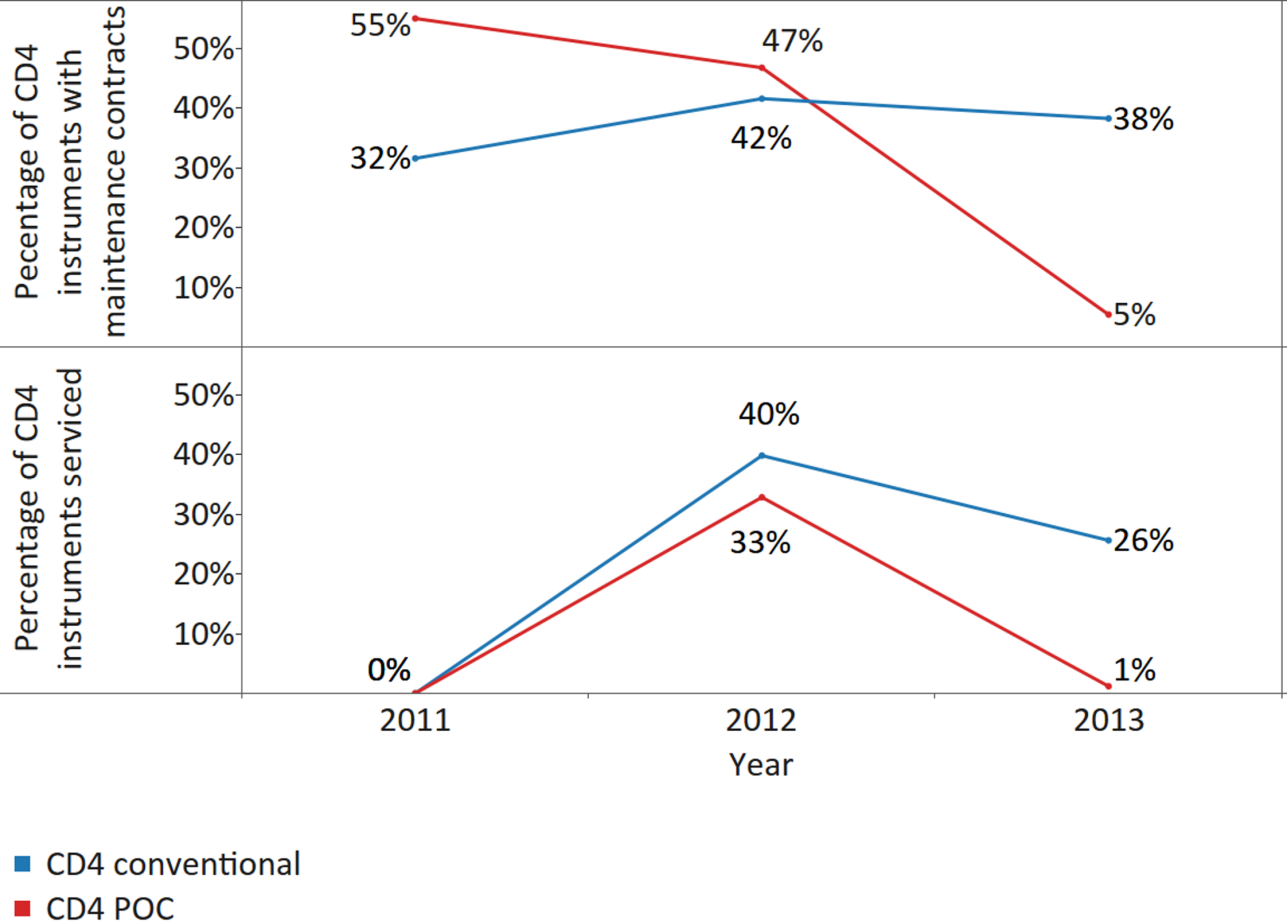
Percentage of CD4 instruments with maintenance contracts (upper panel) and/or serviced (lower panel) in the survey year, disaggregated by CD4 platform, for all reporting countries.

Less than 50% of conventional CD4 instruments were covered with maintenance contracts across all 3 survey years; however, there was an increase in the percentage of instruments reported to be under contract in 2012 and 2013 from 2011 (*X* ^*2*^ [2; 10,672] = 71.4, *p* < 0.001, ES Cramer’s *V* = 0.06) (Fig 7). The proportion of POC CD4 instruments with maintenance contracts dropped significantly from 55% in 2011 to 5% coverage in 2013 (*X* ^*2*^ [2; 3,664] = 953.3, *p* < 0.001, ES Cramer’s *V* = 0.36). The underlying reasons were not investigated; however, the two potential explanations are previously existing maintenance contracts not renewed after expiry and purchasing of more instruments without maintenance contracts. Servicing levels were also low and declined significantly between 2012 and 2013 (conventional CD4, *X* ^*2*^ [1; 7,597] = 174.6, *p* < 0.001, ES *phi* = 0.15; POC CD4, *X* ^*2*^ [1; 2,954] = 629.3, *p* < 0.001, ES *phi* = 0.46): only 26% of conventional CD4 instruments and only 1% of POC CD4 instruments were serviced in 2013 (Fig 7).

### Availability and Use of VL/EID Technology

WHO recommends, in line with the 90-90-90 targets, to perform VL testing 6 mo and 12 mo after ARV treatment initiation and annually thereafter if the patient is stable on ART and to perform at least two EID tests per HIV-exposed infant, including tests at birth, at 6 wk, confirmatory tests, and tests at 9 mo in some countries [5,6]. Data show that countries continue to expand quantitative/qualitative (VL/EID) nucleic acid testing (NAT) capacity to meet WHO recommendations (Table 3).

**Table 3:** Number of VL and EID instruments and available and utilized VL capacity across all reporting countries, disaggregated by WHO region.

WHO Region/Year	Number of VL and EID Instruments*	Available VL Capacity per PLWHA^#^	Available VL Capacity per PLWHA on ART^&^	Number of VL Tests Performed per PLWHA on ART^§^	Number of EID Tests Performed per HIV-Exposed Infant^¶^
**AFRO**					
2011	212	0.2	0.9	0.2	1.1
2012	237	0.3	1.0	0.04	1.4
2013	410	0.3	0.7	0.6	1.1
**AMRO**					
2011	41	2.3	N/A	N/A	1.3
2012	156	1.6	5.1	1.5	1.7
2013	127	1.4	N/A	N/A	1.2
**EMRO**					
2011	27	2.7	26.2	1.3	2.2
2012	42	3.6	32.3	1.8	1.8
2013	40	8.2	37.5	2.0	1.4
**EURO**					
2011	36	1.3	9.5	2.2	2.6
2012	34	2.0	10.0	2.1	2.9
2013	30	1.2	4.7	2.2	1.3
**SEARO**					
2011	77	0.6	4.3	0.9	1.6
2012	73	0.8	3.8	0.9	1.6
2013	107	0.4	1.0	0.2	1.6
**WPRO**					
2011	169	4.7	14.6	0.6	2.5
2012	144	4.5	12.0	0.5	1.4
2013	157	5.9	12.7	0.4	1.2
**Total**					
2011	562	0.5	2.1	0.4	1.2
2012	686	0.7	2.2	0.3	1.4
2013	871	0.4	1.2	0.5	1.2

AFRO, WHO African region; AMRO, WHO Region of the Americas; ART, antiretroviral therapy; EID, early infant diagnosis; EMRO, WHO Eastern-Mediterranean region; EURO, WHO European region; PLWHA, people living with HIV/AIDS; SEARO, WHO South-East Asian region; VL, viral load; WPRO, WHO Western Pacific region.

* Based on a subset of 59, 64, and 60 countries reporting the number of VL instruments in 2011, 2012, and 2013, respectively.

^**#**^ Based on a subset of 54, 61, and 57 countries with data available for number of instruments and number of PLWHA in 2011, 2012, and 2013, respectively.

^**&**^ Based on a subset of 42, 60, and 46 countries with data available for number of VL instruments and number of PLWHA on ART in 2011, 2012, and 2013, respectively.

^**§**^ Based on a subset of 31, 44, and 33 countries with data available for number of VL tests and number of PLWHA on ART in 2011, 2012, and 2013, respectively.

^¶^ Based on a subset of 32, 44, 51 countries with data available for number of EID tests and number of infants in 2011, 2012, and 2013, respectively.

Based on our analysis of 38 countries that reported across all survey years on this survey question, NAT capacity increased by 24.4% between 2011 and 2013 (to 9.7 million tests in 2013 from 7.8 million tests in 2011). Abbott m2000, COBAS AmpliPrep/COBAS TaqMan (48 or 96), COBAS AMPLICOR Analyzer, and NucliSENS EasyQ were the most common instruments on the market, with Abbott m2000 and COBAS AmpliPrep/COBAS TaqMan (48 or 96) expanding their market presence between 2011 and 2013 (Table 4). The survey reveals that technologies that can be used at POC for nucleic acid (VL/EID) testing are not yet being used in reporting countries.

**Table 4:** Market share by number of branded viral load (VL) technologies in 2011, 2012, and 2013 for all responding countries.

VL Technology	WHO PQ Status	2011			2012			2013		
		Number of Reporting Countries	Number of Reported Instruments	Percentage from Total Number of Reported Instruments	Number of Reporting Countries	Number of Reported Instruments	Percentage from Total Number of Reported Instruments	Number of Reporting Countries	Number of Reported Instruments	Percentage from Total Number of Reported Instruments
**Abbott m2000**	Yes	27	104	18.5%	32	191	27.8%	35	255	29.3%
**COBAS AmpliPrep/COBAS Taqman (48 or 96)**	Yes	29	99	17.6%	35	155	22.6%	40	250	28.7%
**COBAS AMPLICOR Analyzer**	No	32	156	27.8%	32	131	19.1%	31	160	18.4%
**NucliSENS EasyQ**	Yes	13	104	18.5%	17	126	18.4%	15	109	12.5%
**VERSANT kPCR Molecular System**	Yes	8	41	7.3%	9	52	7.6%	8	42	4.8%
**Other**	N/A	11	58	10.3%	10	31	4.5%	11	55	6.3%

**Fig 8:**
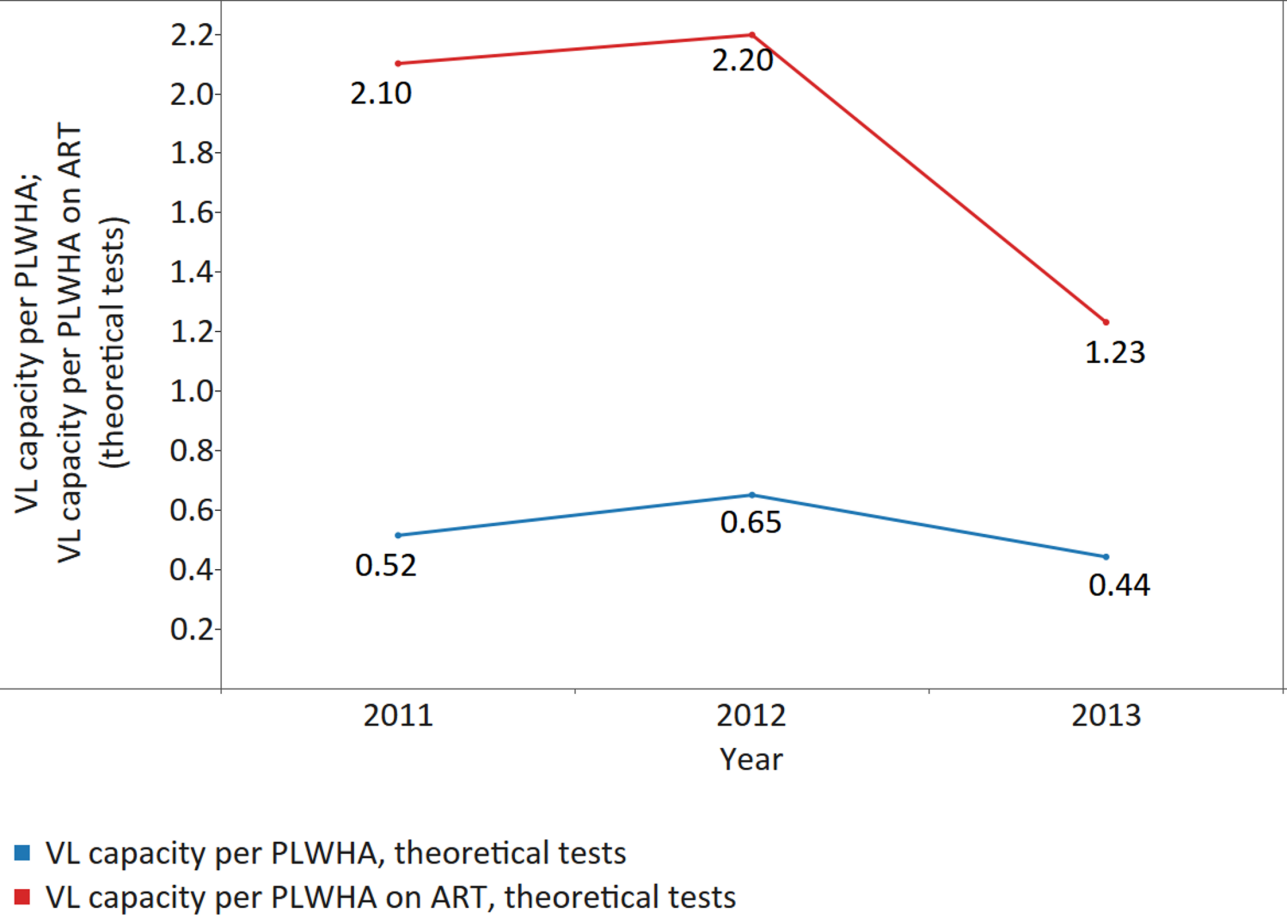
Theoretical viral load (VL) capacity per patient on ART and per PLWHA. Data labels indicate numbers of theoretical tests per year per patient on ART or per PLWHA.

VL instrument capacity is sufficient to cover PLWHA currently on ART but not adequate to cover all PLWHA (Fig 8). The theoretical VL capacity per patient on ART per year was on average 2.10 tests in 2011 (median capacity per patient on ART 5.14; IQR 1.27–17.31; MAD 4.16); 2.20 tests in 2012 (median 4.63; IQR 1.45–11.47; MAD 3.83); and 1.23 tests in 2013 (median 3.59; IQR 0.81–9.12; MAD 2.96) (Fig 8), suggesting that current theoretical VL capacity covers the current needs of PLWHA on ART. Yet, country specific analysis of theoretical VL capacity available revealed that in 2013, 28% of reporting countries did not have adequate VL capacity to perform ≥1 VL test per patient on ART (the theoretical need if all HIV-positive individuals were put on treatment, consistent with the latest WHO recommendations). Theoretical VL capacity per PLWHA (including those who are not on ART) was even lower, reaching only 0.52 tests per y in 2011 (median 0.84; IQR 0.27–2.53; MAD 0.71); 0.65 tests per y in 2012 (median 1.28; IQR 0.35–3.50; MAD 0.99); and 0.44 tests in 2013 (median 0.90; IQR 0.30–2.40; MAD 0.74) (Fig 8). Fifty-four percent of reporting countries did not have the capacity to perform ≥1 VL test per PLWHA per y.

**Fig 9:**
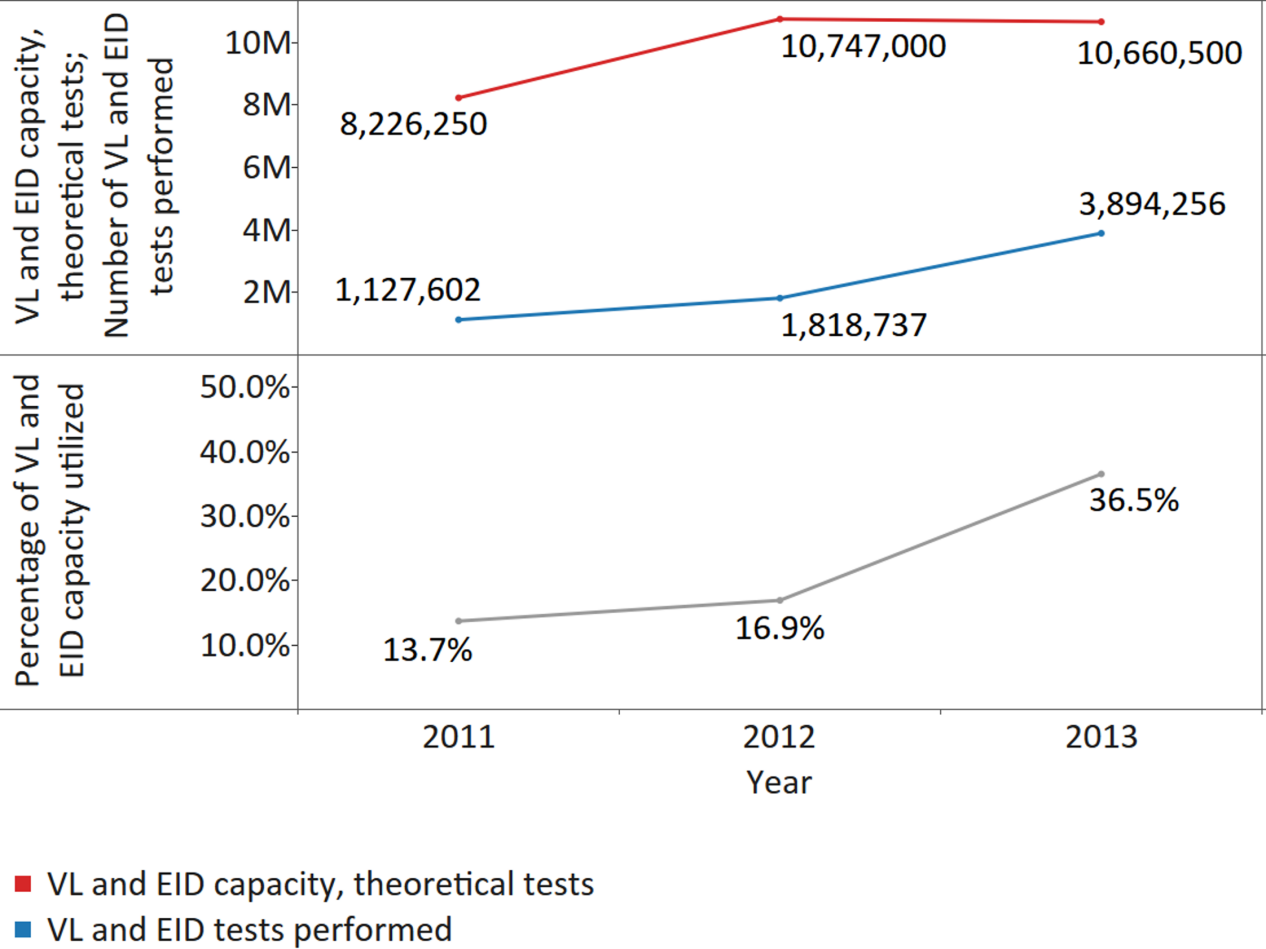
Utilization of viral load (VL)/early infant diagnosis (EID) capacity for all responding countries. The upper panel shows the number of VL/EID tests performed and the theoretical VL/EID capacity. The lower panel shows the percentage of VL/EID capacity utilized during the reporting period.

In addition, utilization of instruments was low, although increasing, throughout the reporting years. Only 13.7% of theoretical capacity was reportedly being used in 2011 (median 7.5%; IQR 2.6%–23.9%; MAD 6.0%); 16.9% (median 11.5%; IQR 2.2%–32.5%; MAD 9.8%) in 2012; and 36.5% (median 9.4%; IQR 2.3%–28.9%; MAD 8.2%) in 2013 (*X* ^*2*^ [2; 29,633,750] = 1,722,577, *p* < 0.001, ES Cramer’s V = 0.17) (Fig 9). Only 25% of 33 reporting countries performed ≥1 VL test per patient on ART in 2013.

**Fig 10:**
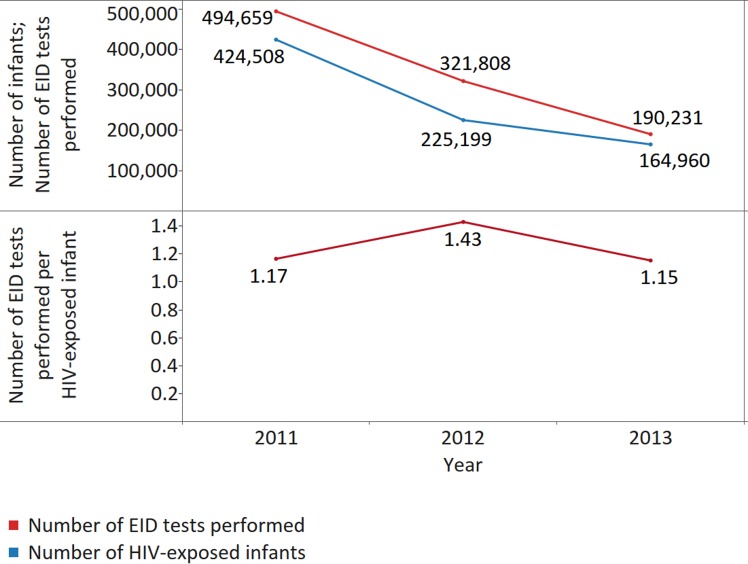
The number of early infant diagnosis (EID) tests performed per infant born to an HIV-positive mother per year for all reporting countries. The upper panel displays the numbers of EID tests performed and the number of infants born to HIV-positive mothers. The lower panel displays the number of EID tests performed per infant born to an HIV-positive mother.

Encouragingly, our data show that across 33, 44, and 51 reporting countries, the number of EID tests performed per infant born to an HIV-positive mother was 1.17 (median 1.10; IQR 1.00–1.91; MAD 0.14) in 2011, 1.43 (median 1.32; IQR 1.01–2.14; MAD 0.32) in 2012, and 1.15 (median 1.20; IQR 1.00–1.94; MAD 0.20) in 2013, suggesting high coverage of EID testing, including confirmatory testing (Fig 10). In 2013, only 3 countries reported less than 1 EID test per infant born to an HIV-positive mother.

**Fig 11:**
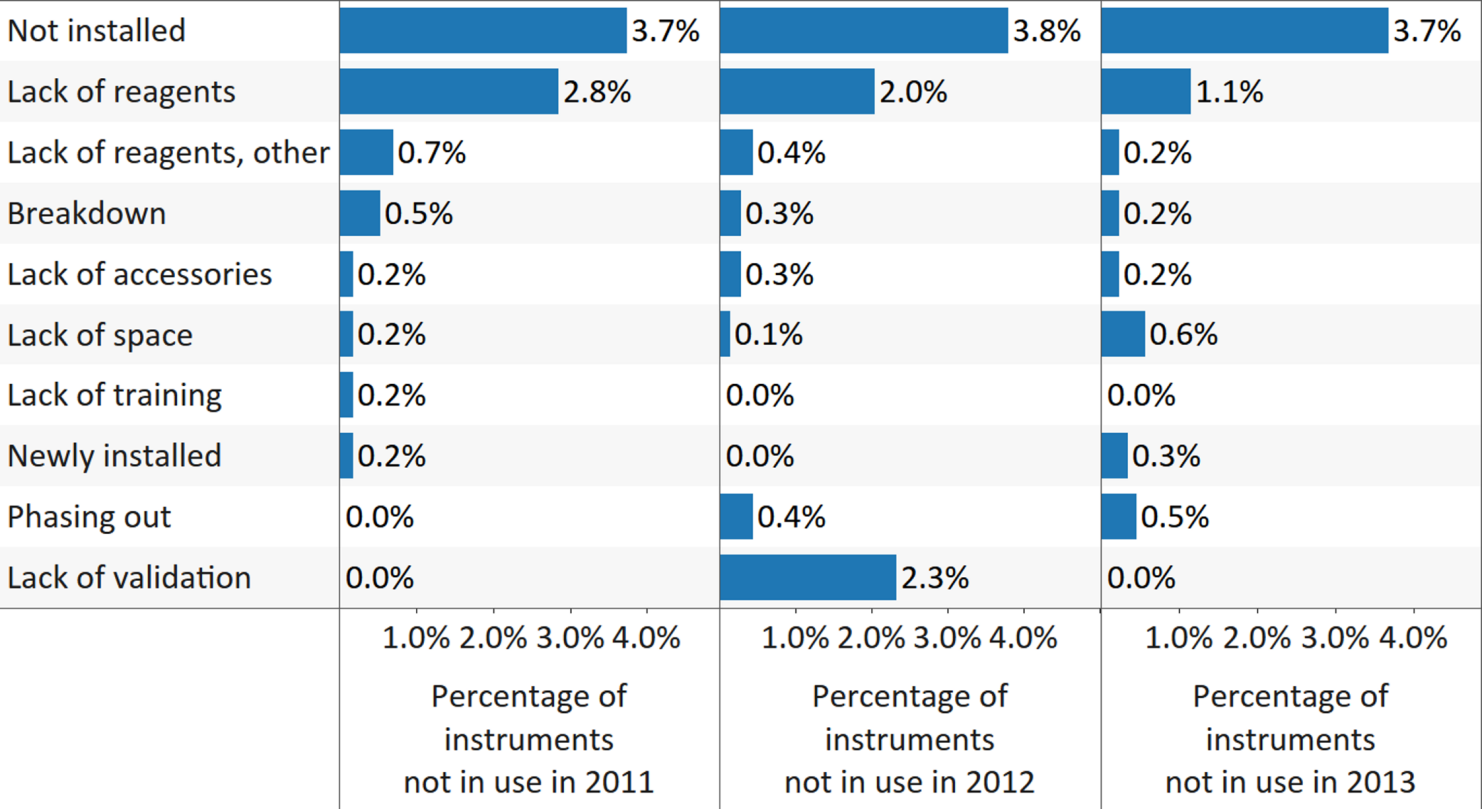
The main reasons for nonutilization of viral load (VL)/early infant diagnosis (EID) instrumentation for all reporting countries. Data labels indicate the percentage from total number of reported instruments in a particular year.

As with survey findings for CD4 instrumentation, the surveys highlighted major issues concerning the functionality and use of VL instruments. Twenty-one (35.6%), 22 (34.4%), and 23 (38.3%) countries reported 12%, 14%, and 10% of all VL/EID instruments nonutilized in 2011, 2012, and 2013, respectively (reporting countries, *X* ^*2*^ [2; 183] = 0.2, *p* = 0.896, ES Cramer’s *V* = 0.02; nonutilized instruments, *X* ^*2*^ [2; 2,119] = 7.7, *p* = 0.022; the difference between 2011 and 2012 and between 2011 and 2013 was not statistically significant, ES Cramer’s *V* = 0.04). Nonutilization was mostly due to noninstallation and a lack of reagents, with no improvements noted over the 3 survey years (noninstallation, *X* ^*2*^ [2; 2,119] = 0.02, *p* = 0.993, ES Cramer’s *V* = 0.002; lack of reagents, *X* ^*2*^ [2; 2,119] = 5.5, *p* = 0.065, ES Cramer’s *V* = 0.04; Fig 11). The number of instruments with maintenance contracts was below 50% across all 3 survey years—increasing from 21% in 2011 to 49% in 2012 and then declining to 38% in 2013 (*X* ^*2*^ [2; 2,119] = 105.8, *p* < 0.001, ES Cramer’s *V* = 0.16). Servicing levels were also low, with 47% of all instruments reportedly serviced in 2012 and 31% in 2013 (*X* ^*2*^ [1; 1,557] = 44.7, *p* < 0.001, ES *phi* = 0.17). No instruments were reported to be serviced in 2011, which may be attributed to a lack of corresponding data at the country level.

## Discussion

Survey results across 3 y demonstrate a trend of increasing numbers of CD4 and VL/EID instruments procured in responding countries. Increasing instrument numbers suggest the expansion of services, particularly at the lower levels of the testing network.

Our data show that for CD4, the number of instruments present in countries is sufficient to meet current demand for not only patients on ART but all PLWHA in the countries surveyed. For VL, data show that while responding countries can theoretically meet demand for testing of PLWHA on ART, current instrument capacity is not yet sufficient to cover all PLWHA: over half (54%) of the responding countries did not have the theoretical VL capacity to perform ≥1 VL test per PLWHA in 2013. These data highlight a lack of capacity in many reporting countries to implement new WHO recommendations [2] on VL testing for treatment monitoring and the need for continued support if new goals are to be achieved.

Despite the fact that many countries have instruments in place and the theoretical capacity to respond to testing needs, our analysis indicates widespread underutilization of CD4 and VL technology. Only 13.7% of existing CD4 capacity was utilized in 2013, with 30.4% of countries performing less than 1 CD4 test per patient on ART per y, which falls short of current recommendations. For VL technology, only 36.5% of theoretical capacity was utilized across reporting countries (median 9.4%; IQR 2.3%–28.9%; MAD 9.8%) in 2013. The stark contrast between available instrument capacity and instrument utilization can be partly explained by the substantial number of machines reported as nonutilized each survey year because of breakdowns, stock outs of reagents, lack of installation, or other reasons (e.g., 7.4% of all CD4 instruments and 10% of VL/EID were not in use by the end of 2013). Another contributing factor, not assessed in these surveys, may be inadequate geographic distribution of CD4 and VL instruments, which may be deployed in low-volume sites, resulting in underutilization of technology. The theoretical capacity should help programme managers and funding agencies to plan and deploy instruments based on their capacity and the volume of tests expected to be done in the laboratory facility. Programme managers and funding partners should not expect 100% capacity utilization: nonetheless, efficient use of available equipment and those to be procured should remain a goal for the national programme managers. Of additional concern are data showing extremely low coverage of instruments with maintenance contracts and infrequent or total absence of servicing for in situ instruments (below 50% for all machines reported across responding countries).

There is a need to assess and address the root causes of instrument underutilization. Lack of reagents, uninstalled and underutilized equipment, maintenance requirements, and staff training are issues that national programme managers and policy makers can and must address. Many of the commonly observed equipment challenges can be addressed and prevented from recurring with site selection and deployment planning that includes timely training and retraining, staff proficiency testing, supply chain strengthening, and enforceable maintenance and servicing contracts. Countries could also explore using equipment lease or reagent rental contracts in which the company provides the machine and the country pays for the reagents. With this scheme, diagnostic companies replace or timely repair a nonfunctioning machine and timely supply reagents as agreed in the contract to ensure that the laboratory activities are not interrupted.

Our findings support the need to strengthen diagnostic capacity in reporting countries [17,18]. Recent data from Médecins Sans Frontières have shown financial constraints as a key reason for incomplete or slow implementation of VL testing [19,20]. Many countries still face numerous implementation and funding shortfalls that make it difficult to put the new WHO guidelines into practice [19,20].

Decentralization of HIV care, in some of the hardest hit countries, will remain difficult unless POC technologies meeting WHO prequalification requirements become available and can be deployed for use in peripheral, low-volume treatment centres [21]. Several POC CD4, POC VL, and POC EID technologies are expected to be available in early 2016 [22,23]. However, POC will not solve all the shortfalls raised by our survey, although it may circumvent the need for trained laboratory specialists at the lower levels of the tiered health system. Regardless of the need for POC, it is clear that laboratory-based monitoring will remain a key component of HIV programmes now and in the future [19,20,24]. While we await new POC VL and POC EID technologies, strengthening of the transport systems that facilitate specimen collection from remote ART facilities (e.g., dried blood spot specimens for EID) must remain a focus.

This is the first attempt to comprehensively gather information on HIV testing and monitoring technology in WHO Member States, with the goal to inform programme managers and funding partners in their effort to increase access to HIV monitoring technologies. Caution needs to be taken when using the results of these surveys because of under-reporting in some national programmes and the potential for reported information to be incomplete. Nevertheless, the response rate had increased by 2013, mitigating responder bias and suggesting a welcome increase in interest in HIV diagnostics. Another important limitation to note is that the survey findings were limited to the public sector. In some countries, the private sector makes an important contribution to HIV treatment and care, and associated diagnostic testing and monitoring will not be reflected in these survey findings. The next step will be to provide guidance and institute data quality control procedures to ensure that comprehensive, validated datasets are available on a yearly basis in order to reduce some of the limitations and inconsistencies that arise when comparing large datasets across multiple countries. WHO will continue to evaluate the market share and performance of laboratory technologies through these annual surveys. Finally, future analyses should consider other potential explanations for variability in laboratory capacity, including level of economic development, donor assistance, and prevalence of HIV and other infections requiring similar laboratory diagnostic approaches, such as viral hepatitis.

Despite significant progress in ART scale-up, which has enabled 16 million people to receive treatment, meeting the UNAIDS 90-90-90 targets depends heavily on the commitment and capacity of governments and international partners to improve access to high-quality testing for EID and treatment monitoring. With laboratory systems in reporting countries expanding, a national laboratory strategic plan to strengthen services must be developed, implemented, and monitored by governments and their national and international partners. The focus of international community, to ensure optimal use of laboratory technologies, should be on those countries where interventions for scaling up access to HIV diagnostic technologies are most needed.

## Supporting Information

S1 Data HIV diagnostic survey data. (XLSX)

S1 Survey Questionnaire WHO/AIDS Medicines and Diagnostics Service (AMDS) survey on the use of antiretroviral (ARV) medicines and laboratory technologies and implementation of WHO related guidelines survey. (DOC)

## Abbreviations

AFRO: WHO African Region
AMDS: AIDS Medicines and Diagnostics Service
AMRO: WHO Region of the Americas
ART: antiretroviral therapy
ARV: antiretroviral
CD4: cluster of differentiation 4
EID: early infant diagnosis
EMRO: WHO Eastern Mediterranean Region
ES: effect size
EURO: WHO European Region
IQR: interquartile range
MAD: median absolute deviation
NAT: nucleic acid testing
NGO: nongovernmental organization
PLWHA: people living with HIV/AIDS
POC: point of care
SEARO: WHO South-East Asia Region
UNAIDS: Joint United Nations Programme on HIV and AIDS
VL: viral load
WHO: World Health Organization
WHO PQ: WHO prequalified
WPRO: WHO Western Pacific Region
