# Supplementary material for: Availability and Use of HIV Monitoring and Early Infant Diagnosis Technologies in WHO Member States in 2011–2013: Analysis of Annual Surveys at the Facility Level
Source: PLoS Med. 2016 Aug 23;13(8):e1002088. doi: 10.1371/journal.pmed.1002088 (PMC4995037; doi:10.1371/journal.pmed.1002088)
Supplement: S1 Survey Questionnaire — (DOC) [file pmed.1002088.s002.doc]

**WHO/AMDS survey on the use of antiretroviral (ARV) medicines and laboratory technologies and Implementation of WHO Related Guidelines**

**Survey to document the situation in [please insert the year]**

The WHO AIDS Medicines and Diagnostics Service (AMDS) of the HIV/AIDS Department is conducting the yearly Survey on Antiretroviral Therapy Regimens and laboratory technologies used in Low- and Middle-income Countries. The questionnaire covers the use of ARVs in adults and children for both antiretroviral therapy (ART) and prevention of mother to child transmission (PMTCT) and the use of laboratory tests for ART initiation and monitoring.

To fill in the questionnaire, please use relevant ART, PMTCT, national laboratory and procurement programmes officers to gather the requested information for the period from **1 January - 31 December [YEAR].** We would appreciate it if the professional officer submitting the data to us could check the **completeness** of the filled questionnaire, the quality, the **accuracy** and the **validity of the information.**

At global and regional levels, the results of this survey will be used to produce regional and global ARV use trend analyses as well as global ARV demand forecasts which will be discussed with ARV and diagnostic manufacturers and donors in order to prevent global shortage.

WHO will analyze the responses of each country to produce country PSM profiles which present country specific strategic information and provide feedback to assist national program managers to develop more cost-effective interventions.

We would be grateful if you could fill in the attached questionnaire and send it back to us : ([kinvie@who.int](mailto:kinvie@who.int)) and ([habiyamberev@who.int](mailto:habiyamberev@who.int))**, by [DATE/ MONTH/YEAR].**

**For any queries concerning this questionnaire, please contact Dr** [**Vincent**](mailto:Vincent) **Habiyambere** ([habiyamberev@who.int](mailto:habiyamberev@who.int))**, HIV/AIDS Department, WHO-Geneva.**

Country: Date completed: _____/_____/_______

Name of person who filled in the questionnaire: ______________________________________

Position: _____________________________________________________________________

Institution:_____________________________________________________________________

E-mail address: _______________________________Phone: __________________________

**SECTION 1A. ARV TREATMENT OVERVIEW**

**Question 1.1.** Number of adults and children on ART at the end of [YEAR] { GARP 4.1}

**Question 1.2.** Number of sites providing ART at the end of [YEAR] { GARP 4.3 }

**SECTION 1B. TREATMENT IN HIV-INFECTED ADULTS AND ADOLESCENTS (≥10 years old) including pregnant women**

**Question 1.** Report the total number of HIV-infected adults and adolescents ≥10 years old by treatment line at the end of [YEAR].

|  | Total number of HIV-infected Adults and adolescents ≥10 years old by treatment line at the end of [YEAR]. |
| --- | --- |
| First line |  |
| Second line |  |
| Third line |  |
| **TOTAL** |  |

**Question 2** Report the number of patients per 1st line ART regimens used in HIV-infected adults and adolescents ≥10 years old at end of [YEAR] including HIV-infected pregnant women who are on ART.

***N.B. Please start by ART regimens with higher numbers by end*** [YEAR].

| List of 1st line ART regimens used in HIV-infected adults and adolescents ≥10 years old regimen at the end of [YEAR]. | Number of HIV-infected adults and adolescents ≥10 years old receiving this ART regimen at the end of [YEAR]. |
| --- | --- |
|  |  |
|  |  |
|  |  |
|  |  |
|  |  |
|  |  |
|  |  |
|  |  |
|  |  |
|  |  |
|  |  |
|  |  |
|  |  |
|  |  |
|  |  |
| **TOTAL** |  |

**Question 3** Report the number of patients per second line ART regimens used in HIV-infected adults and adolescents ≥10 years old at the end of [YEAR].

***N.B. Please start by ART regimens with higher numbers by end*** [YEAR].

| List of 2^nd^ line ART regimens used in HIV-infected adults and adolescents ≥10 years old regimen at the end of [YEAR]. | Number of HIV-infected adults and adolescents ≥10 years old receiving this ART regimen at the end of [YEAR]. |
| --- | --- |
|  |  |
|  |  |
|  |  |
|  |  |
|  |  |
|  |  |
|  |  |
|  |  |
|  |  |
|  |  |
|  |  |
|  |  |
|  |  |
|  |  |
|  |  |
| **TOTAL** |  |

**Question 4** Report the number of patients per third line ART regimens used in HIV-infected adults and adolescents ≥10 years old at end of [YEAR].

***N.B. Please start by ART regimens with higher numbers by end*** [YEAR].

| List of 3^rd^ line ART regimens used in HIV-infected adults and adolescents ≥10 years old at the end of [YEAR]. | Number of HIV-infected adults and adolescents ≥10 years old receiving this ART regimen at the end of [YEAR]. |
| --- | --- |
|  |  |
|  |  |
|  |  |
|  |  |
|  |  |
|  |  |
|  |  |
|  |  |
|  |  |
|  |  |
|  |  |
|  |  |
| **TOTAL** |  |

**SECTION 2. TREATMENT IN HIV-INFECTED CHILDREN (<10 years old)**

**Question 5.** Number of HIV-infected children<10 years old by treatment line at the end of [YEAR].

|  | Total number of HIV-infected children <10 years old by treatment line at the end of [YEAR]. |
| --- | --- |
| First Line |  |
| Second Line |  |
| Third Line |  |
| **TOTAL** |  |

**Question 6.** Report the number of children per 1^st^ line ART regimens used in HIV-infected infants and children <10 years old at the end of [YEAR].

***N.B. Please start by ART regimens with higher numbers by end*** [YEAR].

| List of 1^st^ line regimens used in HIV-infected children at the end of [YEAR]. | Number of HIV-infected infants and children < 10 years old receiving this regimen at the end of [YEAR] by age group | | |
| --- | --- | --- | --- |
|  | # children  **< 3 years old** receiving this regimen **(A)** | # children **≥3 to <10 years old** receiving this regimen **(B)** | Total # children **<10 years old** receiving this regimen  **(A) + (B)** |
|  |  |  |  |
|  |  |  |  |
|  |  |  |  |
|  |  |  |  |
|  |  |  |  |
|  |  |  |  |
|  |  |  |  |
|  |  |  |  |
|  |  |  |  |
|  |  |  |  |
|  |  |  |  |
|  |  |  |  |
|  |  |  |  |
|  |  |  |  |
|  |  |  |  |
| **TOTAL** |  |  |  |

**Question 7.** Report the number of children per second line ART regimen used in HIV-infected children <10 years old at the end of [YEAR].

***N.B. Please start by ART regimens with higher numbers by end*** [YEAR].

| List of 2nd line ART regimen used in HIV-infected children <10 years old at the end of [YEAR]. | Number of HIV-infected children <10 years old receiving this regimen at the end of [YEAR]. |
| --- | --- |
|  |  |
|  |  |
|  |  |
|  |  |
|  |  |
|  |  |
|  |  |
|  |  |
|  |  |
|  |  |
|  |  |
|  |  |
|  |  |
|  |  |
|  |  |
| **TOTAL** |  |

**Question 8.** Report the number of children per third line ART regimen used in HIV-infected children <10 years old at the end of [YEAR].

***N.B. Please start by ART regimens with higher numbers by end*** [YEAR].

| List of 3^rd^ line ART regimen used in HIV-infected children <10 years old at the end of [YEAR]. | Number of HIV-infected children <10 years old receiving this regimen at the end of [YEAR]. |
| --- | --- |
|  |  |
|  |  |
|  |  |
|  |  |
|  |  |
|  |  |
| **TOTAL** |  |

**SECTION 3: PREVENTION OF MOTHER TO CHILD TRANSMISSION**

**Question 9:** Number and % of pregnant women who started antiretrovirals to reduce the risk of mother to child transmission and various PMTCT options during [YEAR] {GARP 3.1}:

**Question 10 :** What is the recommended PMTCT option for HIV-infected pregnant women in your country (please mark (**x**) ):

□ **Option A means:** pregnant HIV+ women not eligible for treatment receive AZT twice daily during pregnancy + single dose-NVP at onset of labor, twice daily maternal AZT +3TC during labor and post-partum for 7 days; extended infant NVP prophylaxis during breastfeeding or short infant prophylaxis if no breastfeeding.

□ **Option B means:** pregnant HIV+ women not eligible for treatment receive triple ARV prophylaxis :AZT+3TC+{LPV/r or ABC or EFV} or TDF+3TC(or FTC)+EFV continued until 1 week after exposure to breastfeeding ends. Short infant ARV prophylaxis added.

□ **Option B+ means:** life time triple ARV therapy for the treatment of all pregnant HIV+ women (regardless of CD4 count) or pregnant HIV+ women meeting eligibility criteria for treatment.

□ Other PMTCT option used in your country (please specify: ____________________________

**Question 11.** Report the number of pregnant women per ARV regimens used in your country for PMTCT Option A in [YEAR].

***N.B. Please start by ARV regimens with higher numbers by end [YEAR].***

| Option A ART regimens used for HIV-infected pregnant women in [YEAR]. | Number of HIV-infected pregnant women who started this regimen in [YEAR]. |
| --- | --- |
|  |  |
|  |  |
|  |  |
| **TOTAL** |  |

**Question 12.** Report the number of pregnant women per ARV regimens used in your country for **PMTCT Option B** in [YEAR].

***N.B. Please start by ARV regimens with higher numbers by end [YEAR].***

| **Option B** ART regimens used for HIV-infected pregnant women in [YEAR]. | Number of HIV-infected pregnant women who started this regimen in [YEAR]. |
| --- | --- |
|  |  |
|  |  |
|  |  |
| **TOTAL** |  |

**Question 13.** Report the number of pregnant women per ARV regimens used in your country for **PMTCT Option B+** in [YEAR].

***N.B. Please start by ART regimens with higher numbers by end [YEAR].***

| Option B+ ART regimens used for HIV-infected pregnant women in [YEAR]. | Number of HIV-infected pregnant women who started this regimen in [YEAR]. |
| --- | --- |
|  |  |
|  |  |
|  |  |
|  |  |
| **TOTAL** |  |

**Question 14.** Report the number of neonates per ARV used in your country for HIV prophylaxis **in neonates born from HIV-infected pregnant women** in [YEAR].

| **ARVs** used for HIV **prophylaxis** of neonates born from HIV-infected mothers in [YEAR]. | Number of neonates started this regimen in [YEAR]. |
| --- | --- |
|  |  |
|  |  |
|  |  |
| **TOTAL** |  |

**SECTION 4: LABORATORY SERVICES**

**HIV tests**

**Question 15.** Total number of HIV tests (RDTs & ELISA) done between 1 Jan- 31 Dec [YEAR]:___

**(Number of people tested for HIV: see GARP)**

**CD4 Tests**

**Question 16.** Total number of CD4 tests done between 1 Jan- 31 Dec [YEAR]:___

**Question 17.** Total number of HIV-infected people who had at least one CD4 test between 1 Jan- 31 Dec [YEAR]: ____

**Question 18.** Total number of patients on ART who had at least one CD4 test between 1 Jan- 31 Dec [YEAR]: ____

**Question 19.** Total number of HIV-infected pregnant women who had at least one CD4 test between 1 Jan- 31 Dec [YEAR]: ____

**Viral load**

**Question 20.** Total number of VL tests done between 1 Jan- 31 Dec [YEAR]: ____

**Question 21.** Total number of all HIV-infected people who had at least one VL test between 1 Jan- 31 Dec [YEAR]: ____

**Question 22.** Total number of patients on ART who had at least one VL test between 1 Jan- 31 Dec [YEAR]: ____

**Question 23.** Total number of HIV-infected pregnant women who had at least one VL test between 1 Jan- 31 Dec [YEAR]: ____

**Early Infant Diagnosis (EID)**

**Question 24.** Total number of EID tests done between 1 Jan- 31 Dec [YEAR]: ___

**Question 25.** Total number of infants (<12 months old) born to HIV-infected mother who had at least one EID test between 1 Jan- 31 Dec [YEAR]: ____

**Question 26.** Report the total number of laboratories (labs) or sites by type of tests in your country

| **Type of laboratory tests** | Total number of labs or sites where samples are collected (sites with testing and sites without testing) by type of test | Total number of labs or sites where the actual testing is done by type of test | Total number of labs or sites where the actual testing is done that participate in an external quality assessment (EQA) scheme by type of test | Total number of labs or sites that need quality improvement activities based on most recent EQA exercise by type of test | List main activities required for quality improvement by type of test |
| --- | --- | --- | --- | --- | --- |
| HIV serology antibody testing including rapid test & ELISA |  |  |  |  |  |
| Early Infant Diagnosis (EID) |  |  |  |  |  |
| CD4 testing |  |  |  |  |  |
| Viral load testing |  |  |  |  |  |
| HIVDR genotype testing |  |  |  |  |  |
| GeneXpert (TB test) |  |  |  |  |  |

**Question 27. Availability of laboratory HIV technologies: Report the number of machines/assays by technology available in your country.**

| **Type of Assay/machine[[1]](#footnote-2)** | Total number of laboratory machines | Number of sites where the lab machine is installed | Total No. of lab machines not in use | **Number of machines not in use by major reason** | | | | | | | Number of equipment with a maintenance service contract | Number of equipment serviced in [YEAR] |
| --- | --- | --- | --- | --- | --- | --- | --- | --- | --- | --- | --- | --- |
|  |  |  |  | No reagents | Not installed | Need repair | No staff trained | Decommissioned | Specify other reason here and No. of machines | Specify the reason here and No. of machines |  |  |
|  |  |  |  |  |  |  |  |  |  |  |  |  |
| **CD4 Technologies** |  |  |  |  |  |  |  |  |  |  |  |  |
| - Alere Pima Analyzer |  |  |  |  |  |  |  |  |  |  |  |  |
| - Apogee Auto40 Flow Cytometer |  |  |  |  |  |  |  |  |  |  |  |  |
| - BD FACSCalibur |  |  |  |  |  |  |  |  |  |  |  |  |
| - BD FACSCount |  |  |  |  |  |  |  |  |  |  |  |  |
| - BD FACSPresto™ Near Patient CD4 Counter |  |  |  |  |  |  |  |  |  |  |  |  |
| - Coulter Epics |  |  |  |  |  |  |  |  |  |  |  |  |
| - Millipore-Guava |  |  |  |  |  |  |  |  |  |  |  |  |
| - Partec CyFlow |  |  |  |  |  |  |  |  |  |  |  |  |
| - Partec miniPOC |  |  |  |  |  |  |  |  |  |  |  |  |
| - PointCare NOW |  |  |  |  |  |  |  |  |  |  |  |  |
| - Other (specify..) |  |  |  |  |  |  |  |  |  |  |  |  |
| **Virological testing Technologies** |  |  |  |  |  |  |  |  |  |  |  |  |
| - Abbott RealTime HIV-1 assay **^(A)^** /manual/m2000rt |  |  |  |  |  |  |  |  |  |  |  |  |
| - Abbott RealTime HIV-1 assay **^(A)^** /m24/m2000rt |  |  |  |  |  |  |  |  |  |  |  |  |
| - Abbott RealTime HIV-1 assay **^(A)^** /m2000sp/m2000rt |  |  |  |  |  |  |  |  |  |  |  |  |
| - Abbott RealTime HIV-1 Qualitative assay **^(B)^** /manual/m2000rt |  |  |  |  |  |  |  |  |  |  |  |  |
| - Abbott RealTime HIV-1 Qualitative assay **^(B)^**  /m2000sp/m2000rt |  |  |  |  |  |  |  |  |  |  |  |  |
| - COBAS® AMPLICOR HIV-1 MONITOR Test **^(A)^** / Amplicor (Roche) |  |  |  |  |  |  |  |  |  |  |  |  |
| - Roche Amplicor HIV-1 DNA test **^(B)^** / Amplicor |  |  |  |  |  |  |  |  |  |  |  |  |
| - COBAS® AmpliPrep/COBAS® TaqMan® HIV-1 **^(A)^** / COBAS TaqMan 48 (Roche) |  |  |  |  |  |  |  |  |  |  |  |  |
| - COBAS® AmpliPrep/COBAS® TaqMan® HIV-1 ^(A)^/ COBAS TaqMan 96 (Roche) |  |  |  |  |  |  |  |  |  |  |  |  |
| - COBAS® AmpliPrep/COBAS® TaqMan® HIV-1 Qualitative **^(B)^** / COBAS TaqMan 48 (Roche) |  |  |  |  |  |  |  |  |  |  |  |  |
| - COBAS® AmpliPrep/COBAS® TaqMan® HIV-1 Qualitative **^(B)^** / COBAS TaqMan 96 (Roche) |  |  |  |  |  |  |  |  |  |  |  |  |
| - GENERIC HIV CHARGE VIRALE **^(A)^** / one NorDiag Arrow instrument |  |  |  |  |  |  |  |  |  |  |  |  |
| - GENERIC HIV CHARGE VIRALE **^(A)^** / two NorDiag Arrow instruments |  |  |  |  |  |  |  |  |  |  |  |  |
| - NucliSENSEasyQ® HIV-1 **^(A)^** / NucliSens miniMAG / EasyQ® (bioMerieux) |  |  |  |  |  |  |  |  |  |  |  |  |
| - NucliSENSEasyQ® HIV-1 **^(A)^** / NucliSens easyMAG / EasyQ® (bioMerieux) |  |  |  |  |  |  |  |  |  |  |  |  |
| - VERSANT® HIV-1 RNA 1.0 Assay (kPCR) (**^A)^** / VERSANT® kPCR Molecular System (Siemens) |  |  |  |  |  |  |  |  |  |  |  |  |
| - Please list below other virological technologies found in your country but not reported above (specify..) |  |  |  |  |  |  |  |  |  |  |  |  |
|  |  |  |  |  |  |  |  |  |  |  |  |  |
|  |  |  |  |  |  |  |  |  |  |  |  |  |
| **^(A)^** - Assay intended to be used for measuring levels of HIV-1 RNA (viral load)  **^(B)^** - Assay intended for qualitative detection of HIV-1 RNA and DNA in adult and pediatric (including younger than 18 months of age: EID) patients. | | | | | | | | | | | | |

**SECTION 5: COUNTRY TARGETS**

**Question 28.** In the table below, report the national targets for ART, PMTCT and lab tests in the next 5 years

| **Country target** | **At the end of** [YEAR] | **At the end of** [YEAR] | **At the end of** [YEAR] | **At the end of** [YEAR] | **At the end of** [YEAR] |
| --- | --- | --- | --- | --- | --- |
| ***1. Number of adults and children to be on ART*** |  |  |  |  |  |
| **Subset 1.1:** Number of adults and adolescents (≥10 years) to be on ART |  |  |  |  |  |
| **Subset 1.2:** Number of children <10 years to be on ART |  |  |  |  |  |
| **Sub-subset 1.2.1:** Number of children <5 years to be on ART |  |  |  |  |  |
| **Sub-subset 1.2.2:** Number of children ≥ 5 to <10 years to be on ART |  |  |  |  |  |
| ***2. Total Number of pregnant women who started ART for PMTCT*** |  |  |  |  |  |
| **Subset 2.1:** Number of pregnant women on Option B+ |  |  |  |  |  |
| **Subset 2.2:** Number of pregnant women on Option B |  |  |  |  |  |
| **Subset 2.3:** Number of pregnant women on Option A |  |  |  |  |  |
| ***3. Total number of people who will be tested for HIV infection*** |  |  |  |  |  |
| ***4. Total number of people who will have CD4 tested*** |  |  |  |  |  |
| ***5. Total number of people who will have VL tests*** |  |  |  |  |  |
| ***6. Total number of children (born from HIV infected women) who will have EID tests*** |  |  |  |  |  |
| ***7. Total number of HIV serology tests*** |  |  |  |  |  |
| ***8. Total number of CD4 tests*** |  |  |  |  |  |
| ***9. Total number of VL tests*** |  |  |  |  |  |
| ***10. Total number of EID tests*** |  |  |  |  |  |

1. This list of technologies is updated annually to insert newly WHO pre-qualified technologies and/or technologies reported by countries in previous survey year. [↑](#footnote-ref-2)
